# Effect of organic tomato (*Lycopersicon esculentum*) extract on the genotoxicity of doxorubicin in the *Drosophila* wing spot test

**DOI:** 10.1590/S1415-47572009005000008

**Published:** 2009-01-17

**Authors:** Elaine S. Dutra, Cristina D. Dias, Bethânia C. de Araújo, Antônio J. S. Castro, Júlio C. Nepomuceno

**Affiliations:** Laboratório de Mutagênese, Instituto de Genética e Bioquímica, Universidade Federal de Uberlândia, Uberlândia, MGBrazil

**Keywords:** antioxidants, co-genotoxicity, *Drosophila melanogaster*, organic tomato, SMART

## Abstract

The consumption of organic tomatoes (ORTs) reduces the risk of harmful effects to humans and the environment caused by exposure to toxic agrochemicals. In this study, we used the somatic mutation and recombination test (SMART) of wing spots in *Drosophila melanogaster* to evaluate the genotoxicity of ORT and the effect of cotreatment with ORT on the genotoxicity of Doxorubicin^®^ (DXR, a cancer chemotherapeutic agent) that is mediated by free radical formation. Standard (ST) cross larvae were treated chronically with solutions containing 25%, 50% or 100% of an aqueous extract of ORT, in the absence and presence of DXR (0.125 mg/mL), and the number of mutant spots on the wings of emergent flies was counted. ORT alone was not genotoxic but enhanced the toxicity of DXR when administered concomitantly with DXR. The ORT-enhanced frequency of spots induced by DXR may have resulted from the interaction of ORT with the enzymatic systems that catalyze the metabolic detoxification of this drug.

## Introduction

Pesticides have been used increasingly in agriculture, and today more than 1,000 chemicals are classified as pesticides. Because large quantities of these chemicals are released into the environment every day and many of them affect non-target organisms, they pose a potential risk to human health (Zeljezic and Garaj-Vrhovac, 2001). Ren *et al.*, (2001) demonstrated that some vegetable varieties cultivated without pesticides and with organic fertilizer offer more protection against several mutagenic compounds than cultivated vegetables in general. According to Abreu and Stoltenborg (1998), organic agriculture is a production system committed to health, ethics, and the preservation of nature. This system makes intelligent use of natural resources, traditional methods and the most advanced ecological technologies.

Doxorubicin hydrochloride (Doxorubicin^®^; DXR) is a potent antitumor agent that is used to treat various types of human cancer because of its capacity to genetically damage tumor cells from laboratory animals and humans (Gentile *et al.*, 1998). The antitumor activity of DXR is mediated its ability to stimulate the formation of a variety of free radical species (Keizer *et al.*, 1990). Using *Drosophila melanogaster* as a test organism, Costa and Nepomuceno (2006) found that DXR induces mutations in the somatic mutation and recombination test (SMART) assay and that antioxidant vitamins (vitamins C, E, and β-carotene) and polyminerals (copper, selenium, and zinc) protect against the genotoxicity of this drug.

Nutritional therapy with antioxidants administered concomitantly with antineoplasic drugs offers several benefits in the treatment of cancer patients. The use of antioxidant vitamins such as A, E, and C mitigates the side effects associated with antiblastic drugs and has a beneficial effect on the course of treatment since the toxicity of antineoplastic drugs is a limiting factor in this type of therapy. Antioxidant-based nutritional therapy can therefore be a valuable adjuvant during oncological therapy by improving the control of cancer (Santos and Cruz, 2001). In this context, the consumption of tomatoes and tomato products is considered a nutritional indicator of good eating habits and a healthy life style because of the presence of different antioxidant molecules such as carotenoids, particularly lycopene, ascorbic acid, vitamin E and phenol compounds, particularly flavonoids (George *et al.*, 2004).

In this study, we used the *D. melanogaster* wing spot test to examine the effects of an extract of agrochemical-free organic tomato (ORT) on the genotoxicity of DXR.

**Figure 1:**
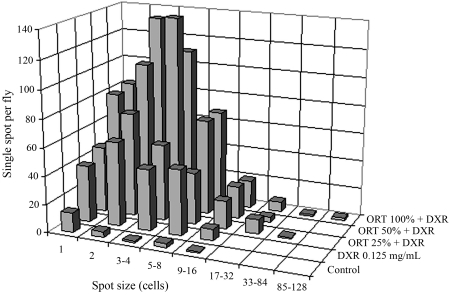
Size distributions for single spots after chronic treatment with different concentrations of organic tomato extract (ORT) and Doxorubicin^®^ (DXR). Larvae from standard (ST) cross.

## Materials and Methods

### Chemicals

Doxorubicin^®^ ((8S-cis)-10-[(3-amino-2,3,6,-trideoxy-alpha-lyxohexapyranosyl)oxy]-7,8,9,10-tetrahydro-6,8,11-trihydroxy-8-(hydroxyacetyl)-1-ethoxy5,12 naphthalenedione; DXR; MW 580.0; C_27_H_29_NO_11_.HCl) (CAS 23214-92-8) was supplied by Laboratório Eurofarma Ltda. (São Paulo, SP, Brazil) and was used at a concentration of 0.125 mg/mL. Each bottle supplied by the manufacturer contained 10 mg of lyophilized DXR.

### Preparation of organic tomatoes

Organic tomatoes were donated by Santa Teresa do Alto farm (Itupeva, SP, Brazil). The fresh fruit was washed, weighed, made into a juice, and aqueous extracts were prepared as described by Ito *et al.* (1986). Briefly, the fruits were processed in a domestic blender (Black & Decker^®^ SB30T) and the juice was filtered through gauze to obtain pure extract (100% ORT extract). Water was added to 100% ORT extract obtained from 100 g of fruit to provide dilutions containing 25% and 50% of extract.

### Stock strains of *Drosophila melanogaster*

The somatic mutation and recombination detection test (SMART) on *Drosophila melanogaster* wing cells was used to determine the genotoxicity of ORT extract alone and in association with DXR. The mutant strains of *D. melanogaster* (*flr*, flare; *mwh*, multiple wing hairs; *TM3*, Third Multiple3 Beaded Serrate) were supplied by Dr. Ulrich Graf of the Institute of Pharmacology and Toxicology, University of Zurich, Schwerzenbach, Switzerland. The strains were kept in 250 mL jars containing *Drosophila* culture medium (820 mL water, 25 g biological yeast (*Saccharomyces cerevisiae*), 11 g agar, 150 g banana, and 1 g nypagin).

Virgin *flr*^*3*^*/In(3 LR)TM3, ri p*^*p*^*sep l(3)89 Aa bx*^*34e*^ and *Bd*^*s*^ females were crossbred with *mwh/mwh* males, referred to as the standard (ST) cross (Graf *et al.*, 1996). This cross was used to generate flies with marked heterozygotes (MH: *mhw* + / + *flr*^*3*^).

### Egg collection

Eggs from the ST offspring were collected over an 8 h period and placed in flasks containing solid agar (3% agar in water) and a layer of *S. cerevisiae* supplemented with saccharose. After 72 ± 4 h the larvae were washed in distilled water and collected with a fine mesh sieve.

### Experimental procedure

Third-stage ST larvae were placed in glass jars (2.5 cm diameter x 8.0 cm high) containing 1.5 g of instant potato purée medium (HIKARI^®^; São Paulo, SP, Brazil) and 5 mL of aqueous ORT extract, with or without DXR (0.125 mg/mL). A positive control was treated with DXR alone, and a negative control was treated with distilled water. The larvae were exposed for approximately 48 h, during which time they moved up the walls of the jars as they progressed to the pupal stage.

### Microscopic analysis

Emerging adults with the genotype *mwh* +/+ *flr*^*3*^ were collected and preserved in 70% ethanol. The wings were removed from the flies by using entomological pincers and an Olympus stereoscopic microscope, and then mounted on slides and covered with coverslips using Faure solution (30 g gum arabic, 20 mL glycerol, 50 g chloral hydrate, 50 mL water). The dorsal and ventral sides of the wings were examined with an Olympus light microscope at 400X magnification to identify single (*flr*^*3*^ or *mwh* phenotype) or twin (*flr*^*3*^ and *mwh* phenotypes) clones. The size of the clones or spots (number of cells affected) was recorded, as was the type of clones (single *mwh* or *flr*^*3*^ and twin clones), since there is a correlation between the time of induction of the genetic lesions and the final size of the clones (Graf *et al.*, 1984).

### Statistical analysis

Statistical analysis of the genotoxicity of ORT was done using the test described by Frei and Würgler (1988). For treatment combinations, the Mann-Whitney-Wilcoxon *U* test (Frei and Würgler, 1995) was used to compare the frequency of each type of spot per fly in the treated flies compared with the negative control, and the frequency of spots in ORT + DXR flies compared to flies treated with DXR alone. A value of p ≤ 0.05 indicated significance.

## Results and Discussions

Table 1 shows the frequencies of mutant spots in the trans-heterozygote markers (MH) of ST flies, the larvae of which had fed on ORT extract, DXR or different concentrations of aqueous ORT extract with DXR. Feeding on medium containing up to 100% ORT did not significantly increase in any class of spots (single small, single large, twin and total spots) when compared with the negative control. In contrast, DXR (0.125 mg/mL) significantly increased the frequency of all classes of spots (p = 0.05). The increase in twin spots indicated that DXR was recombinogenic. The recombinogenic activity of DXR in somatic cells of *D. melanogaster* has also been reported by Lehmann *et al.* (2003), Rodriguez-Arnaiz *et al.* (2004) and Costa and Nepomuceno (2006). Concomitant treatment with ORT extract and DXR significantly increased the frequencies of single small, single large, twin and total spots in larvae fed medium containing 50% or 100% ORT extract plus DXR than in larvae fed medium containing DXR alone; the increases with medium containing 25% ORT extract were not significant (Table 1).

The increases in spot frequency indicated that ORT extract potentiated the genotoxicity of DXR. This potentiation interfered with the relationship between the formation of clones (*mwh* and *flr*^*3*^) and the age of the developing fly. Imaginal discs are tissues that grow continually by mitotic division throughout larval development. At the beginning of development, the discs that make up the wings consist of 50-100 cells and reach approximately 25,000 cells by the beginning of the pupal stage, when wing differentiation begins (Graf, 1995). Continual cellular proliferation during larval development leads to an increase in the number of target cells in the imaginal disc. In contrast, the size of the induced clones is expected to diminish as the age at which they are induced increases.

There is a clear connection between the time of induction, the frequency of spots and the size of the mutant clones. Few spots are formed in young larvae because the target cell population is small, but they are generally large in size because they have time to expand; in contrast, the frequency of spots in older larvae is considerably higher but the spots are smaller (Costa and Nepomuceno, 2003). As shown here, the ORT-mediated potentiation of DXR genotoxicity resulted in a larger number of spots. Frei and Würgler (1996) reported that large mutant cell clones induced by camptothecin (a DNA topoisomerase inhibitor) can result from the merger of small contiguous clones. This possibility was also discussed by Torres *et al.* (1998), Nepomuceno (Nepomuceno JC, PhD thesis, Universidade de Brasília, Brasília, DF, Brazil, 1999) and Valadares (Valadares BLB, MSc dissertation, Universidade Federal de Uberlândia, Uberlândia, MG, Brazil, 2002). Hence, co-genotoxicity can increase the frequency of small and large clones.

There was an increase in large spots containing 17-32, 33-84 and 85-128 cells in the descendants of larvae treated concomitantly with ORT and DXR (Figure 1). This distribution of large spots was not seen in the descendants of larvae treated only with DXR. This finding indicated that co-treatment with ORT and DXR increased the genotoxicity of DXR and allowed the emergence of a greater number of large and small mutant spots, as proposed by Frei and Würgler (1996).

The mechanisms whereby ORT increases the genotoxicity of DXR were not analyzed directly here. However, the major DXR metabolite is doxorubicinol, produced via a cytosolic carbonyl reductase-catalyzed reduction of the ketone at C-13 of the parent drug. The main enzyme implicated in this reduction is NADPH:cytochrome P450 reductase, a flavoprotein that catalyzes the one electron reduction of DXR to the semiquinone radical; the latter is cytotoxic in hypoxic environments through its ability to covalently modify cellular macromolecules. Under aerobic conditions, the semiquinone radical undergoes redox cycling, leading to the generation of reactive oxygen species (ROS) such as superoxide anion, hydrogen peroxide and hydroxyl radical. The resulting oxidative stress contributes to the cytotoxicity of DXR and is particularly important in the dose-limiting cardiotoxicity associated with this and other anthracyclines (Riddick *et al.*, 2005). Interestingly, cytochrome P450 also plays a role in a DXR detoxification pathway involving reductive deglycosylation to a 7-deoxyaglycone metabolite (Niitsu *et al.*, 2000).

Based on these observations, one possible mechanism of action for the ORT extract could be via interaction with the enzyme systems that catalyze the metabolic detoxification of DXR. Cytochrome P450 enzymes may be inhibited by the ORT extract, thereby reducing the detoxification of DXR. Lehmann *et al.* (2000) hypothesized a similar mechanism to account for an increase in the frequency of mutant spots in *D. melanogaster* larvae co-treated with tannic acid (a phenolic compound found in foods), methylmethanesulfonate and nitrogen mustard. Tomatoes contain phenolic compounds (Peng *et al.*, 2008). Teel and Huynh (1998) reported that the enzymatic activity of cytochrome P450 can be altered by phytochemicals and that phytochemicals can affect the metabolism of substrates for these enzymes. Many vegetables and fruits are important inhibitors of cytochrome P450 (Farhan and Cross, 2002; McFadyen *et al.*, 2004; Girennavar *et al.*, 2007), primarily through their content of phenolic compounds (Lehmann *et al.*, 2000).

Lycopene, a carotenoid with potent antioxidant properties (Heber and Lu, 2002), occurs in many fruits and vegetables, with high concentrations in tomatoes. Tomatoes are also rich in vitamins, particularly provitamin A (735-1270 UI/100 g), as well as vitamin C or ascorbic acid (15-23 mg), vitamin B1 and riboflavin or vitamin B2 (Amara-Mokrane *et al.*, 1996). These vitamins interfere with free radicals and other reactive metabolites generated by DXR, thereby decreasing the genotoxicity of this drug (Amara-Mokrane *et al.*, 1996; Antunes and Takahashi, 1998; Costa and Nepomuceno, 2006; Fragiorge *et al.*, 2007). However, the complex chemical composition of tomatoes (Peng *et al.*, 2008) means that the component(s) responsible for enhancing the mutagenic and recombinogenic activity of DXR remain to be identified.

In conclusion, aqueous extracts of organic tomato contain one or more components capable of potentiating the genotoxicity of DXR. Additional studies are required to identify the tomato component(s) responsible for this activity.

## Figures and Tables

**Table 1:** Frequency of mutant spots observed in standard (ST) cross trans-heterozygote markers of *D.melanogaster* treated with doxorubicin (DXR) in the absence and presence of organic tomato extract (ORT).

Series^ ^			Spots per fly (n. of spots); stat. diagnoses^a^					
DXR (mg/mL)	ORT (%)		N. of flies	Small single spots (1-2 cells)^b^	Large single spots (> 2 cells)^b^	Twin spots	Total spots	Spots with *mwh* clone ^c^
0	0		40	0.45 (18)	0.13 (05)	0.05 (02)	0.63 (25)	23
0	25		40	0.30 (12) ns	0.13 (05) ns	0.05 (02) ns	0.48 (19) ns	19
0	50		40	0.48 (19) ns	0.05 (02) ns	0.05 (02) ns	0.58 (23) ns	23
0	100		40	0.45 (18) ns	0.10 (04) ns	0.03 (01) ns	0.58 (23) ns	23
0.125	0		40	2.40 (96)	1.73 (69)	1.43 (57)	5.55 (222)	200
0.125	25		40	2.78 (111) ns	2.00 (80) ns	1.63 (65) ns	6.40 (256) ns	243
0.125	50		40	4.40 (176)*	4.23 (169)*	3.05 (122)*	11.68 (467)*	418
0.125	100		40	5.10 (204)*	3.15 (126)*	3.00 (120)*	11.25 (450)*	425

^a^Statistical diagnoses according to Frei and Würgler (1995) with a two-tailed *U*-test. *p ≤ 0.05; ns, not significant. ^b^Including rare *flr*^*3*^ single spots. ^c^Considering *mwh* clones from single and twin *mwh* spots.
